# How do free healthcare policies impact utilization of maternal and child health services in fragile settings? Evidence from a controlled interrupted time series analysis in Burkina Faso

**DOI:** 10.1093/heapol/czae077

**Published:** 2024-08-24

**Authors:** Thit Thit Aye, Hoa Thi Nguyen, Laurène Petitfour, Valéry Ridde, Felix Amberg, Emmanuel Bonnet, Mariam Seynou, Joël Arthur Kiendrébéogo, Manuela De Allegri

**Affiliations:** Heidelberg Institute of Global Health, Medical Faculty, University of Heidelberg, Im Neuenheimer Feld 130.3, Heidelberg 69120, Germany; Heidelberg Institute of Global Health, Medical Faculty, University of Heidelberg, Im Neuenheimer Feld 130.3, Heidelberg 69120, Germany; Heidelberg Institute of Global Health, Medical Faculty, University of Heidelberg, Im Neuenheimer Feld 130.3, Heidelberg 69120, Germany; Centre Population et Développement (Ceped), Institut de Recherche pour le Développement (IRD) et Université Paris Cité, Inserm ERL 1244, 45 Rue Des Saints-Pères, Paris 75006, France; Heidelberg Institute of Global Health, Medical Faculty, University of Heidelberg, Im Neuenheimer Feld 130.3, Heidelberg 69120, Germany; Seine saint Denis, Institut de Recherche pour le Développement, 5, cours des humanités, Aubervilliers Cedex F-93 322, France; UMR, 215 Prodig, 5, cours des Humanités, Aubervilliers Cedex F-93 322, France; Service Scientifique et Technique, Centre de Recherche en Santé de Nouna (CRSN)/ Institut National de Santé Publique (INSP), Nouna Secteur No. 6 Rue Namory KEITA, Nouna Po Box: 02, Burkina Faso; Heidelberg Institute of Global Health, Medical Faculty, University of Heidelberg, Im Neuenheimer Feld 130.3, Heidelberg 69120, Germany; Department of Public Health, University Joseph Ki-Zerbo, 04 BP 8398, Ouagadougou 04, Ouagadougou, Burkina Faso; Department of Public Health, Institute of Tropical Medicine, Nationalestraat 155, Antwerp 2000, Belgium; Department of Health Research, Recherche pour la Santé et le Développement (RESADE), 04 BP 8398, Ouagadougou 04, Ouagadougou, Burkina Faso; Heidelberg Institute of Global Health, Medical Faculty, University of Heidelberg, Im Neuenheimer Feld 130.3, Heidelberg 69120, Germany

**Keywords:** Free healthcare policy, healthcare service utilization, facility-based delivery, child health, Burkina Faso

## Abstract

Burkina Faso has implemented a nationwide free healthcare policy (*gratuité*) for pregnant and lactating women and children under 5 years since April 2016. Studies have shown that free healthcare policies can increase healthcare service use. However, the emerging coronavirus disease 2019 pandemic, escalating insecurity and the political situation in recent years might have affected the implementation of such policies. No studies have looked at whether the *gratuité* maintained high service use under such changing circumstances. Our study aimed to assess the effects of *gratuité* on the utilization of facility-based delivery and curative care of children under 5 years in light of this changing context. We employed a controlled interrupted time series analysis using data from the Health Management Information System and annual statistical reports of 2560 primary health facilities from January 2013 to December 2021. We focused on facility-based deliveries and curative care for children under 5 years, with antenatal care and curative care for children over 5 years as non-equivalent controls. We employed segmented regression with the generalized least square model, accounting for autocorrelation and monthly seasonality. The monthly utilization rate among children under 5 years compared to those above 5 years (controls) immediately increased by 111.19 visits per 1000 children (95% CI: 91.12, 131.26) due to the *gratuité*. This immediate effect declined afterwards with a monthly change of 0.93 per 1000 children (95% CI: −1.57, −0.29). We found no significant effects, both immediate and long-term, on the use of maternal care services attributable to the *gratuité*. Our findings suggest that free healthcare policies can be instrumental in improving healthcare, yet more comprehensive strategies are needed to maintain healthcare utilization. Our findings reflect the overall situation in the country, while localized research is needed to understand the effect of insecurity and the pandemic at the local level and the effects of *gratuité* across geographies and socioeconomic statuses.

Key messages

- Free healthcare policies can be a suitable strategy for ensuring access to healthcare in the context of the COVID-19 pandemic and the socio-political instability in Burkina Faso.
- The most recent national free healthcare policy did not increase the number of women delivered in health facilities immediately or over time due to the well-established previously national fee-subsidized policy (Soins Obstétricaux et Néonataux d’Urgence).
- In contrast, the policy increased healthcare use among children under 5 years immediately after its implementation, but its effect declined over time.
- Free healthcare alone is not enough to maintain healthcare utilization. A more comprehensive strategy is needed to expand service coverage and overcome health system challenges.

## Introduction

Long-term and sustainable investments in healthcare are difficult in low- and middle-income countries, especially in fragile settings (WHO, 2023). In these settings, it is crucial to prioritize essential health services that ensure maximum coverage and financial protection. In the past two decades, advocates promoted eliminating user fees in Africa through free healthcare policies, which target essential health services such as maternal and child health (Ridde, 2015). Free healthcare policies have been successful in increasing service utilization (Lagarde and Palmer, 2008; Ridde *et al*., 2011; Leone *et al*., 2016). However, if not carefully planned and implemented, this approach can lead to funding and supply shortages, increased healthcare workers workload and reduced service quality (Lagarde and Palmer, 2008; Ridde *et al*., 2012). Concerns about the sustainability of free healthcare highlight the need for a comprehensive approach to maintaining high-service utilization (Witter and Adjei, 2007; Touré, 2015; Obare *et al*., 2018; Mathonnat *et al*., 2020).

During conflicts, individuals are vulnerable to injuries and infectious diseases, yet accessing healthcare services is often hindered by insecurity and financial constraints (Martineau *et al*., 2017; Bendavid *et al*., 2021). Free healthcare is considered a suitable strategy for those facing financial challenges to access healthcare in such circumstances (Martineau *et al*., 2017). However, implementing such policies is challenging given the harm inflicted upon medical facilities, inadequate resources and a shortage of skilled professionals resulting from conflicts (Martineau *et al*., 2017; Munyuzangabo *et al*., 2021).

Alongside ongoing conflicts, the coronavirus disease 2019 (COVID-19) pandemic emerged as a significant barrier, posing challenges to health systems. Across 105 surveyed countries, essential health services were disrupted, with only 14% providing fee exemptions despite collective efforts from the World Health Organization and governments (WHO, 2020). In Africa, the pandemic caused both delays in people seeking healthcare and also disruptions in healthcare provision (Adu *et al*., 2022). In fragile settings, the pandemic exacerbated challenges in accessing healthcare, depleting resources from maternal and child health services and disrupting childhood immunization (Rodo *et al*., 2022).

**Figure 1.**
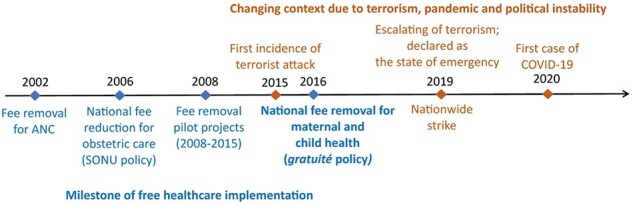
Timeline of free healthcare policies with recent evolving situations in Burkina Faso

Burkina Faso has been investing in free healthcare for the past two decades (Figure 1) (Ridde and Bicaba, 2009; Meda *et al*., 2019). However, recent challenges include political instability, frequent terrorist attacks and the pandemic, which intensified strain on the healthcare system (Makoni, 2022). Insecurity led to health facilities closures and reduced health services, hindering access to necessary healthcare (Makoni, 2022). Preliminary evidence suggests that terrorist attacks in Burkina Faso reduced antenatal care visits and facility-based deliveries (Druetz *et al*., 2020). The pandemic further disrupted health services, affecting malaria treatment and maternal and child health (Médecins Sans Frontières (MSF), 2020; Assefa *et al*., 2021).

Burkina Faso launched a policy called *gratuité* in a few selected districts in April 2016, which was expanded nationwide in June 2016 (Meda *et al*., 2019), providing free healthcare to all pregnant and lactating women and children under 5 years. This policy was motivated by the success of its predecessor, the national fee reduction policy for obstetric care (Soins Obstétricaux et Néonataux d’Urgence [SONU]) and pilot free healthcare projects (Zombré *et al*., 2017; 2019; Nguyen *et al*., 2018; Meda *et al*., 2023). *Gratuité* allows free access to primary, secondary and tertiary services. Several studies have examined the effect of *gratuité* on service usage, and they generally agree that *gratuité* has led to an immediate increase in the utilization of child healthcare services (Ouédraogo *et al*., 2020; Sia *et al*., 2020; Debe *et al*., 2022; Ilboudo and Siri, 2023; Offosse *et al*., 2023). However, its effect on maternal healthcare services, such as antenatal and facility-based delivery, has been insignificant (Ilboudo and Siri, 2023; Offosse *et al*., 2023).

The existing evidence on the effects of *gratuité* in Burkina Faso, however, relies on data collected before 2019 (Ouédraogo *et al*., 2020; Sia *et al*., 2020; Debe *et al*., 2022; Ilboudo and Siri, 2023; Offosse *et al*., 2023). This means that existing literature mostly captures the short-term effects of *gratuité* and cannot adequately capture long-term effects in light of a changing context due to the pandemic and increasing political instability. Moreover, previous studies encountered at least one of the following methodological limitations: first, the non-nationwide scope limited generalizability (Sia *et al*., 2020; Offosse *et al*., 2023); second, the absence of control groups affected the establishment of causal relationships (Sia *et al*., 2020; Ilboudo and Siri, 2023); third, interpreting incidence rate ratios as outcomes may be challenging for decision-makers because maternal and child healthcare utilization is usually reported as a proportion or rate (Debe *et al*., 2022; Ilboudo and Siri, 2023).

Our study expanded the research timeframe to include data up to 2021, encompassing 5 years following the introduction of *gratuité*. This expansion allowed us to examine the policy effects in light of the evolving context in Burkina Faso. Additionally, the use of interrupted time series analysis (ITSA) with independent controls helped us distinguish the policy effects from other events happening during the same period, such as the health worker strike taking place between April and November in 2019 (Bonkoungou, 2020), the COVID-19 pandemic, terrorist attacks and the overall climate of socio-political insecurity. While we expect these events to have affected both the intervention and the control groups, we expect the *gratuité* to have affected only the intervention groups. Hence, our analytical approach helped us to minimize the risk of history bias from concurrent events. Our study aimed to assess both the short-term and long-term effects of *gratuité* on the utilization of facility-based delivery for women and curative care for children under 5 years in Burkina Faso between 2016 and 2021.

## Materials and methods

### Study setting

Burkina Faso, with a 22.1 million population (The World Bank, 2021b), has a gross domestic product (GDP) per capita of 888.8 USD (The World Bank, 2021a), and around 40% of its population lives below the poverty line (The World Bank, 2018). The country spent 6.72% of its GDP on health, primarily funded by the government, external funds and out-of-pocket expenditures (WHO, 2021).

Since 2015, attacks by Jihadist insurgents have displaced millions, exacerbating political instability (Blake, 2019). The country faced additional challenges, including a national health worker strike in 2019 (Boxshall *et al*., 2020) and the COVID-19 pandemic in 2020 (Assefa *et al*., 2021), intensifying pressures on the healthcare system.

The public health system operates across three levels, encompassing 70 health districts across 13 regions as of 2021. Primary health facilities, Centre de Santé et de Promotion Sociale (CSPS) and Centre Médical (CM), at the district level, provide essential healthcare services, including pregnancy and facility-based delivery. Centre Médical avec Antenne chirurgicale (CMA) serves as a district referral centre for surgeries and Caesarean sections. Secondary and tertiary health facilities provide advanced healthcare at the regional and central levels.

Between 2015 and 2019, the service coverage index of maternal and child health slightly increased, from 57 to 60 out of 100 (WHO and World Bank, 2021). The maternal mortality ratio in 2021 was 198 per 100 000 live births (INSD et ICF, 2023), and the under-five mortality rate was 48 per 1000 live births (INSD et ICF, 2023).

### Study intervention: the gratuité policy

The Ministry of Health implemented the *gratuité* policy to improve the financial accessibility of essential healthcare for women and children under 5 years. Fully funded by the government without external assistance, the policy substitutes user fees with government payments to health facilities, employing pre-determined fixed rates for services to beneficiaries (Ministère de la santé du Burkina Faso and Ministère de l’économie des finances et du développement, 2018; Meda *et al*., 2019).

### Study design

We adopted an ITSA with independent controls to assess the *gratuité* effect. Given its nationwide implementation, we could not find equivalent controls. Replicating Meda’s approach (Meda *et al*., 2023), we identified services not targeted by *gratuité* that could serve as non-equivalent controls. We used the first antenatal care visit (ANC1) as a comparison for facility-based delivery. Both ANC1 and facility-based delivery share similar socioeconomic factors and benefits during the same period (Meda *et al*., 2023), but ANC services have been free since 2002 (Ridde and Bicaba, 2009). We used curative care for children over 5 years as a comparison to care for children under 5 years. We chose this group because children over 5 years are not exempted from paying fees and their pre-policy utilization trend is similar to that of children below 5 years. We analysed 9 years of monthly data from January 2013 to December 2021. Given that the *gratuité* was rolled out at the national level in June 2016, we set June 2016 as the interruption point and divided the overall observation period into two segments: a pre-policy period until May 2016 (41 months) and a post-policy period from June 2016 onwards (67 months). This long time series allowed us to measure both the immediate and long-term effects of the policy and be interpreted in light of Burkina Faso’s changing context.

### Outcomes and controls

Table 1 summarizes outcome variables and their respective controls, as defined in our study. Aligned with the focus of *gratuité*, we examined two outcomes: (1) the percentage of facility-based deliveries in primary health facilities including CSPS, CM and CMA and (2) the rate of curative care visits for children under 5 years per 1000 children of the same age group. These outcomes are compared with their respective controls: (1) the percentage of ANC1 visits and (2) the rate of curative care visits for children aged 5–14 years per 1000 children of the same age group.

**Table 1.** Variables and their measurements

	Variable name	Type	Definition
1	Facility-based delivery	Outcome	Total number of uncomplicated deliveries, complicated deliveries and C-sections in the reported month as a percentage of average monthly expected deliveries
	ANC1	Control	Total number of ANC1 visits in the reported month as a percentage of average monthly expected pregnancies
2	Curative care for children aged <5 years	Outcome	Total new cases of curative care visits for children aged <5 years divided by the total population of children aged <5 years, expressed per 1000 children
	Curative care for children aged 5–14 years	Control	Total new cases of curative care visits for children aged 5–14 years divided by the total population of children aged 5–14 years, expressed per 1000 children

### Data sources and data set-up

We retrieved data from two sources: The Health Management Information System (HMIS, the primary data source) and the Ministry of Health’s annual statistical reports.

#### HMIS

We obtained monthly counts of service utilization from 2560 health facilities across all 70 health districts, including CMA, CM and CSPS. We identified monthly outliers for each facility using a modified *Z*-score (Malembaka *et al*., 2021). During data management, we identified ∼7% missing values for each outcome, attributed to a national strike in 2019, which led to data gaps in certain facilities from June to October 2019. We imputed missing values through local polynomial regression for each facility with up to 10 consecutive gaps. This imputation method is well-suited for our complex time series, in which each facility’s data show different patterns (Pérez-González *et al*., 2009; You *et al*., 2016).

#### The Ministry of Health’s annual statistical reports

We gathered information on estimated pregnancies, deliveries, total population and population in different age groups of the 70 health districts from annual statistical reports between 2013 and 2020. According to the annual statistical reports, population estimates from these years were based on the 2006 census, while the 2021 annual report used 2019 census data to estimate population data for the first time. To be consistent with the previous years, we used the linear extrapolation method to estimate population data for 2021. We then calculated average monthly estimates by dividing annual estimates by 12.

#### Preparation of variables

Since our study focused on the overall policy effects, we formed a monthly time series at the national level in two steps. First, monthly counts of service use at the facility level were aggregated by districts and transformed into percentages and rates using appropriate denominators at the district level. Second, district-level percentages and rates were averaged to form national-level percentages and rates for analysis.

### Analytical approach

We inspect trends, seasonality and functionality by plotting outcome and control variables separately. Next, we calculated summary statistics for overall, pre-policy and post-policy periods. To address autocorrelation, we conducted the Durbin–Watson test with 12 lags (Jandoc *et al*., 2015) and plotted the autocorrelation function and partial autocorrelation function to identify autoregression (AR) and moving average (MA) processes (Shin, 2017). With identified AR and MA processes, we employed a generalized least squares (GLS) model using maximum-likelihood estimation to address autocorrelation terms. We observed seasonal patterns in the graphs and included a monthly factor to address seasonal changes (Bhaskaran *et al*., 2013; Shin, 2017). We compared the Akaike Information Criteria (AIC) values of these tested models for model fitness and selected the best models (i.e. models with lowest values) (Supplementary Appendix B).

Additionally, we tested linear and quadratic functional forms to determine trends and selected the best model through the AIC and likelihood ratio test. For facility-based delivery, we included AR and MA processes at the 12th lag (*p* = 12, *q* = 0), with additional quadratic terms for the post-policy trend. Child care analysis involved processes with linear form at the second and first lag (*p* = 2, *q* = 1). Our final models are as follows:

Equation 1: Final GLS model for facility-based delivery:


(1) $$\begin{array}{l}{\rm Facility - based\, delivery} = \beta 0 + \beta {\rm 1time} + \beta {\rm 2level} + \beta {\rm 3trend} + \\ \beta 4 {\rm trendsq} + \beta {\rm 5delivery} +\beta {\rm 6delivery *time} + \beta {\rm 7delivery*} \\ level + \beta {\rm 8delivery*trend} + \beta {\rm 9delivery*trendsq} + {\rm S} + \varepsilon \end{array}$$

We defined interrupted time series components for intervention and control using a standard procedure (Wagner *et al*., 2002). The variable ‘time’ represents months from January 2013 to December 2021, ranging from 1 to 108. It serves as a functional term for pre-policy trends. ‘Level’ distinguishes the pre-policy period (0) from the post-policy period (1) and assesses immediate changes after policy implementation. ‘Trend’ represents the monthly effect after implementation, with values of 0 for the pre-policy period and 1–67 for the post-policy period. ‘Trendsq’ represents quadratic terms for the post-policy trend. The binary variable ‘delivery’ differentiates between the facility-based delivery (1) and ANC1 (0). The dummy variables ‘delivery × time’, ‘delivery × level’, ‘delivery × trend’ and ‘delivery × trendsq’ are interaction terms of facility-based delivery with the previously mentioned variables.

In the above equation, coefficients *β*_0_ to *β*_4_ represent the control group (ANC1), where *β*_0_ is the initial level, *β*_1_ represents the pre-policy trend, *β*_2_ indicates the immediate (level) change in post-policy implementation, *β*_3_ represents the monthly change (trend) in post-policy and *β*_4_ is the quadratic trend. Coefficients *β*_5_ to *β*_9_ represent the intervention group (facility-based delivery), where *β*_5_ represents the level difference between delivery and ANC1 before the policy, *β*_6_ indicates the trend difference between delivery and ANC1 prior to the policy, *β*_7_ represents the immediate level change in delivery compared to the ANC1 in post-policy, *β*_8_ is the monthly changes (trend) in delivery compared to ANC1 following policy implementation and *β*_9_ is the quadratic changes in delivery compared to ANC1 in post-policy. Lastly, ‘*S*’ represents the seasonality.

Equation 2: Final GLS model for curative care for children:


(2) $$\begin{array}{l}{\mathrm{Child\_healthcare}} = \beta 0 + \beta {\rm 1time} + \beta {\rm 2level} + \beta {\rm 3trend} + \\ \beta {\rm 4U5} + \beta {\rm 5U5*time} + \beta {\rm 6U5*level} +\beta {\rm 7U5*trend} + S + \varepsilon\end{array}$$

The second equation resembles the first but focuses on children. Coefficients *β*_0_ to *β*_3_ represent factors influencing children over 5 years, while *β*_4_ to *β*_7_ represent relative differences in healthcare services use between children below 5 years and those over 5 years.

We used model outputs to determine absolute and relative differences between predicted and counterfactual values annually, from the first year after policy implementation up to the fifth year. To calculate the counterfactual values, we assumed that the observed difference between the intervention and control groups would have remained consistent if the policy had not been implemented.

We conducted sensitivity analyses to ensure model accuracy. This involved running a single ITSA for each outcome variable, introducing the interruption in April 2016 instead of June, using ANC4 as a control, excluding 5 months in 2019 with substantial missing data and running models without 2021 data that used imputed values for denominators.

We used STATA 17 BE for data management and RStudio for the analysis. We performed statistical tests at a significance level of 0.05.

## Results

### Summary statistics

In Table 2, we report the summary statistics on the monthly utilization of maternal and child health services from January 2013 to December 2021.

**Table 2.** Summary statistics

	Entire period		January 2013 to May 2016		June 2016 to December 2021	
Indicator	Mean	SD^b^	Mean	SD^b^	Mean	SD^b^
Facility-based delivery (%)	76.80	8.42	78.07	6.67	76.02	9.3
ANC1 (%)	78.63	7.55	81.93	7.32	76.62	7.01
Curative care for children <5 years (rate^a^)	195.97	71.56	135.72	48.72	232.85	56.81
Curative care for children 5–14 years (rate^a^)	37.50	21.80	34.05	11.8	39.62	25.96

a Rate per 1000 children.

b SD = standard deviation.

Before the *gratuité*, about 78% of deliveries and 82% of ANC1 happened in health facilities. After the *gratuité*, deliveries and ANC1 in health facilities slightly declined (76.02% and 76.62% respectively) compared to the pre-policy period.

Before *gratuité*, 136 out of 1000 children under 5 years visited health facilities, four times higher than children over 5 years (34.05/1000 children). After the *gratuité*, health visits increased for both groups, but more pronounced for children under 5 years (232.85 vs 39.62/1000 children).

### The effect on facility-based delivery for women

**Figure 2.**
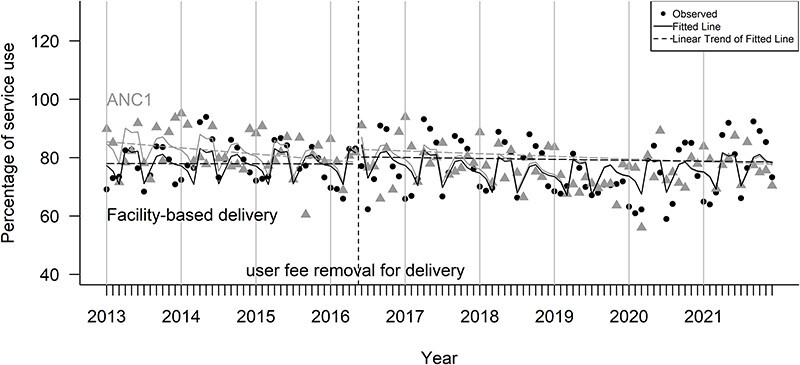
Monthly utilization of facility-based delivery vs ANC1 in Burkina Faso (2013–21)

Figure 2 shows the monthly utilization rates of facility-based delivery and ANC1 over time, with a vertical line indicating the *gratuité* implementation. The plotted points represent observed values, whereas the curvy lines represent fitted lines obtained from model estimation. Trends for facility-based delivery and ANC1 remain similar across the period.

Table 3 provides the selected model estimates for facility-based delivery compared to antenatal care before and after *gratuité*. Initially, nearly 85% of women used ANC1 service and about 77% delivered their babies in facilities. Both service types showed similar trends before *gratuité*. After *gratuité*, we observed no significant changes in the ANC service use. Likewise, we found no significant changes in the use of facility-based deliveries immediately or overtime.

**Table 3.** Model estimation of the gratuité effect on the utilization of facility-based delivery

Facility-based delivery (ANC1 as control)	Estimates (%)	95% CI	*P*-value^a^
**Initial level**			
ANC1 (*β*_0_)	84.9	78.25, 91.55	<0.001***
Difference between facility-based delivery and ANC1 (*β*_5_)	−7.65	−14.19, −1.1	0.023 *
**Pre-policy trend**			
ANC1 (*β*_1_)	−0.18	−0.36, 0.01	0.061
Difference between facility-based delivery and ANC1 (*β*_6_)	0.17	−0.09, 0.43	0.193
**Post-policy period (ANC1)**			
Level (*β*_2_)	4.8	−1.24, 10.83	0.121
Trend (*β*_3_)	−0.25	−0.66, 0.17	0.249
Trend (square) (*β*_4_)	0.01	0,0.01	0.049 *
**Effect of *gratuité* (facility-based delivery)**			
Level change (*β*_7_)	−2.1	−10.6,6.39	0.628
Trend change (*β*_8_)	−0.1	−0.69,0.49	0.740
Trend change (square) (*β*_9_)	−0.0003	−0.01, 0.01	0.926

a *** p-value <0.001, ** p-value <0.01, * p-value <0.05.

Table 5 presents estimated absolute and relative differences in the percentage of facility-based deliveries compared to the counterfactual values (i.e. the values if *gratuité* had not been implemented) in the post-policy period. In the first year after the *gratuité* implementation, the number of women who have their babies delivered in facilities increased by 0.99% compared with its counterfactual value. However, this increase decelerated over time, with a relative decline of 4.6% in the fifth year.

### The effect on curative care for children under 5 years

**Figure 3.**
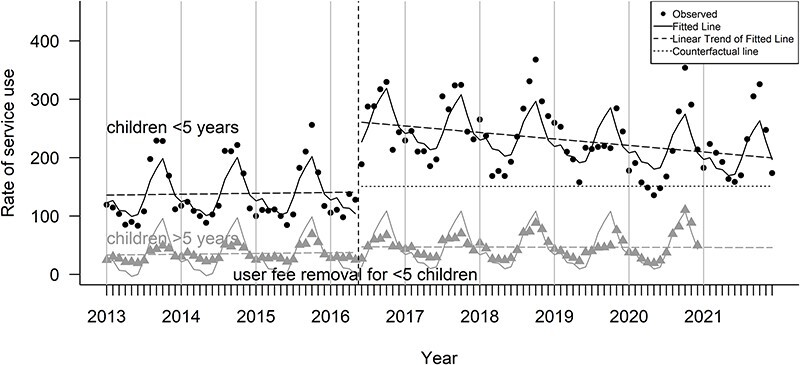
Monthly utilization of curative care for children aged <5 years vs 5–14 years in Burkina Faso (2013–21)

Figure 3 visualizes the utilization of children under 5 years and over 5 years. Pre-*gratuité* trends were similar for both age groups. Immediately after *gratuité*, the utilization rate increased substantially for children under 5 years, but remained the same for those over 5 years. Following the initial increase, we observe a slight, but steady decline in utilization rates for children under 5 years until the end of 2021.

Table 4 shows the results of the selected model for children under 5 years. Roughly 20 out of 1000 children over 5 years sought cares at health facilities initially, whereas roughly 123 (20.25 + 102.54) out of every 1000 children under 5 years visited the facilities. The pre-policy trend remained the same for both age groups. Following the policy implementation, approximately 111 more children under 5 years visited health facilities attributable to the policy. However, after this initial rise, the number of visits declined slightly by 1 per 1000 children each month until the end of the study period.

**Table 4.** Model estimation of the gratuité effect on the utilization of curative care for children <5 years

Curative care for children <5 years (children 5–14 years as control)	Estimates (rate)^b^	95% CI	*P*-value^a^
**Initial level**			
Children 5–14 years (*β*_0_)	20.25	4.01, 36.48	0.015 *
Difference between children <5 years and 5–14 years (*β*_4_)	102.54	87.07, 118	<0.001 ***
**Pre-policy trend**			
Children 5–14 years (*β*_1_)	0.11	−0.38, 0.59	0.664
Difference between children <5 years and 5–14 years (*β*_5_)	0.02	−0.66, 0.7	0.950
**Post-policy period (children 5–14 years)**			
Level change (*β*_2_)	9.56	−5.48, 24.59	0.215
Trend change (*β*_3_)	−0.12	−0.58, 0.33	0.594
**Effect of *gratuité* (children <5 years)**			
Level change (*β*_6_)	111.19	91.12, 131.26	<0.001 ***
Trend change (*β*_7_)	−0.93	−1.57, −0.29	0.005 **

a *** p-value <0.001, ** p-value <0.01, * p-value <0.05.

b Rate per 1000 children.

Table 5 shows that in the first year of policy implementation, the number of children under 5 years who visited health facilities was around 43% higher than expected if the policy had not been in place. However, this percentage decreased annually, reaching roughly 14% by the fifth year after implementation.

**Table 5.** Absolute and relative effects of the policy on maternal and child health service utilization

Indicator	Year	Predicted	Counterfactual	Absolute change	Relative change (%)
Facility-based delivery (%)	Year 1 (June 2017)	81.05	80.26	0.79	0.99
	Year 2 (June 2018)	79.03	79.59	−0.55	−0.70
	Year 3 (June 2019)	78.38	80.37	−2	−2.49
	Year 4 (June 2020)	79.09	82.63	−3.54	−5.99
	Year 5 (June 2021)	81.17	86.34	−5.17	−4.60
Curative care for children aged <5 years (per 1000 children)	Year 1 (June 2017)	215.95	150.59	65.36	43.40
	Year 2 (June 2018)	204.85	150.65	54.21	35.98
	Year 3 (June 2019)	193.76	150.7	43.05	28.57
	Year 4 (June 2020)	182.66	150.76	31.9	21.16
	Year 5 (June 2021)	171.56	150.82	20.74	13.75

### Robustness check

We tested the robustness of our findings using the sensitivity analyses mentioned in our method session. All different sensitivity analyses were consistent with our chosen models. For facility-based deliveries, all sensitivity analyses showed no significant immediate or long-term changes following the policy implementation (Supplementary Appendices A, C–E and G). As for children under 5 years, all models yielded similar immediate effects, with slight variations in the magnitude of coefficients. The long-term effect of all models showed a declining trend, with the model with quadratic terms and the model with an interruption in April showing insignificant effects (Supplementary Appendices A, C, F and H).

## Discussion

Our paper contributes to the existing literature by providing valuable insights into the long-term effects of *gratuité* in Burkina Faso on maternal and child health service utilization. Importantly, our study assesses the extent to which the policy was resilient amidst the country’s recent instability, driven by the combined effects of the COVID-19 pandemic and increased political insecurity resulting from terrorist threats.

Two main findings emerged from the analysis. First, we did not observe any significant changes, both immediately and over time, in the monthly utilization of facility-based delivery following *gratuité* implementation. Second, we detected an immediate and significant increase in healthcare utilization for children under 5 years after *gratuité* implementation. After the initial substantial increase, the utilization rate steadily declined until December 2021.

The absence of significant changes in the utilization rate of facility-based delivery may be explained by the pre-existence of the SONU policy from 2006 until the start of *gratuité* policy. The SONU policy had already subsidized up to 80% of user fees for facility-based delivery. The impact of user fee exemption might have reached its limit due to the effects of the previous policy, suggesting that solely eliminating financial barriers may not be sufficient to bridge existing gaps. Policymakers may need strategies addressing additional barriers, such as transportation and quality of service delivery (De Allegri *et al*., 2011). Besides, the effect of *gratuité* may vary regionally, being more effective in areas with higher poverty concentrations or lower service utilization (Nguyen *et al*., 2018). This existence of the former policy may also explain the insignificant effect of *gratuité* in the long run. Moreover, the pre-existing and continuous drug shortages may lead some women to pay out of pocket for obstetric care (Banke-Thomas *et al*., 2023). This financial burden could hinder certain women from accessing healthcare, delaying progress in expanding coverage for maternal care services.

Our findings on the strong and immediate effects on child healthcare reflect prior findings by Zombré *et al*. (2017) and Nguyen *et al*. (2018), demonstrating the immediate effect of user fee removal on utilization of essential healthcare services. However, our finding of a gradual decline in healthcare use after the initial rise differs from the results of the two studies, which focused on pilot projects in the Sahel region. These projects, supported by international institutions and funds, experienced smoother implementation with fewer challenges (Bierschenk, 2014). In contrast, our nationwide evaluation of a government-managed and financed programme may face more resource allocation and management challenges (Petticrew *et al*., 2005). Our nationwide focus in this study limited us from identifying regional and socioeconomic variations in effects. Further research is needed to investigate how policy effects may vary under different contextual factors. Lastly, differences in policy implementation times may lead to varying magnitudes of effects. While previous policies like SONU and pilot projects might have encountered fewer challenges in the past, *gratuité* implementation may have faced more difficulties due to increasing instability caused by conflicts and COVID-19 in recent years.

We hypothesize that the following reasons could explain the decline of the effect of the *gratuité* on child healthcare utilization after the deep rise observed immediately following the introduction of the policy. First, the initial announcement of the nationwide free healthcare policy might have encouraged very high healthcare seeking, possibly resulting in a high level of healthcare utilization during the early implementation phases. It is possible to imagine that lifting financial barriers encouraged all caregivers to take their children to healthcare facilities, even those who did not suffer from an acute illness episode, but who might have long foregone care due to the imposition of user charges. It is very plausible to imagine that after this initial phase, healthcare utilization gradually adjusted over time. Second, as part of the *gratuité* policy, the government invested in strengthening community-based care. Community-based care is delivered by community health workers who focus on health awareness and curative care for common childhood illnesses such as malaria, diarrhoea and respiratory conditions. A parallel analysis of reimbursement data (data not shown) indicates that the quantity of resources the government channelled towards community care has increased over time. It reflects the higher demand for care to be provided at the community level without the need to travel to the health facility for treatment of simple conditions.

Third, we also acknowledge that constraints on the health budget might have created an implementation gap, causing delays in reimbursement and drug shortages in health facilities, especially so in late 2018 and 2019 (Boxshall *et al*., 2020; Banke-Thomas *et al*., 2023). Recent developments in Burkina Faso’s socio-political circumstances might escalate reimbursement challenges over time. Some children may have to pay for curative care out of pocket due to reimbursement delays and drug shortages (Banke-Thomas *et al*., 2023), imposing new access barriers. Lastly, we note that changes in morbidity patterns of childhood diseases may also lead to reduced health service utilization. However, we lack sufficient epidemiological data to verify this assumption. In any case, we recognize that although all the aforementioned explanations may contribute to the decline of child health service utilization, their effects may be implausible to separate. Further qualitative inquiry is needed to examine the veracity of these hypotheses. In contrast, we note here that we cannot explain the decline observed over time in relation to events such as the COVID-19 pandemic and/or conflict since those have affected both intervention and control groups. Our estimation models indicate instead the presence of a decline specific to the intervention group, i.e. children under 5 years.

Although our method could isolate the *gratuité* effect from the wider socio-political challenges affecting the country, we cannot ignore the increasing political instability within the country and its impact on the health system. Conflicts in Burkina Faso displaced 560 000 people in 2019, which increased to almost 1.6 million in 2021 (UNHCR, 2019; 2021). This has not only decreased facility-based deliveries but also affected over 400 health facilities, leading to the closure of around one-third of them by 2021 (Druetz *et al*., 2020; Makoni, 2022). Makoni also highlights attacks on health workers, exacerbating challenges within the health system. Additionally, ongoing political instability may shift the country’s focus from health to national security. When the health sector faced budget shortages in 2018–19 (Boxshall *et al*., 2020; Banke-Thomas *et al*., 2023), the 2020 defence budget surpassed the health budget by tenfold (UN Women, 2022). This budget shift might exacerbate the drug shortages, leading to further reductions in health service utilization and possibly higher out-of-pocket expenditure. Future research should explore how conflict affects the supply and demand of healthcare services, considering specific health system indicators.

In addition, the COVID-19 pandemic might have posed additional challenges to the health system. The pandemic has caused delays and reductions in maternal and child health services (Adu *et al*., 2022), but the extent of its impact on health service usage in the country remains unclear. Further research can focus on understanding the impact of the pandemic on healthcare utilization in Burkina Faso.

## Methodological considerations

In our analysis, we employed an ITSA with independent controls (i.e. ANC1 as a control for facility-based deliveries and consultations of children above 5 years as a control for children under 5 years). This approach allowed us to analyse the effects of a national policy like *gratuité* while minimizing the potential influence of other concurrent events, reducing history bias. Yet, we acknowledge several limitations in our study. First, we utilized observational routine data from HMIS, which can introduce potential confounding or bias from other uncontrolled interventions or events, such as other interventions, coinciding with *gratuité*. To enhance internal validity, we integrated non-equivalent controls into our analysis. The use of non-equivalent controls accounts for historical bias only to a given extent, particularly due to the impact of COVID-19, which can affect different health services in different ways. To mitigate this bias, we conducted various sensitivity analyses to examine and control for other events occurring during the same period (Supplementary Appendices E–H). Additionally, we conducted sensitivity analyses accounting for the effects of the COVID-19 period (Supplementary Appendices G and H). The results showed that the effect of COVID-19 is not statistically significant. However, we acknowledge that the impact of COVID-19 in Burkina Faso has not been fully explored, and our primary goal is to predict the effect of *gratuité*. Therefore, the findings regarding the impact of COVID-19 should be interpreted with caution. Second, conflict-afflicted areas may have experienced disruptions in HMIS reporting, impacting data quality. We addressed this issue through data imputation and purposely excluding 2022 data due to a high number of missing incidents. Third, being key locations for complicated deliveries and caesarean sections, the absence of facility-based delivery data from regional and central-level hospitals may underestimate facility-based delivery utilization. Fourth, using ANC1 as control may correlate with facility-based delivery, but with ANC being free since 2002 and ANC1 utilization exceeded 90% for over a decade (INSD et ICF, 2012), the risk of bias is low. We conducted a sensitivity analysis to confirm the ANC trend. Fifth, population-based estimates suggest higher facility-based deliveries and antenatal care (INSD et ICF, 2012; 2023) compared to our findings. We used data from annual statistical reports, based on Burkina Faso’s 2006 census data, considered somewhat outdated. However, as both facility-based deliveries and ANC1 relied on this same data source, the impact on our estimates is minimal. Sixth, variations in drug shortages may impact delivery care differently than ANC1, as well as consultations for children under 5 years compared to those over 5 years. These distinctions between outcomes and controls could influence our estimation. Seventh, the use of imputed missing data for 2019 may overestimate and potentially lead to biased results. However, we minimized this risk by conducting sensitivity analyses, and the results showed similar results from the main model. Last, we acknowledge that children who have recently turned 5 years may still benefit from fee exemptions (Banke-Thomas *et al*., 2023), potentially leading to overestimating effects. However, the existence of a strict verification system suggests that this scenario likely applies to only a few children.

## Conclusion

Our study confirmed that *gratuité* immediately increased health service utilization rates among children under 5 years and maintained the rates achieved by the SONU policy for obstetric care services. Although the free healthcare in Burkina Faso has demonstrated its capacity to result in increases in health service utilization, we detected declining utilization among children in the long term. This decline indicates remaining barriers to accessing healthcare, including resource constraints, transportation and service quality. Research should investigate health system challenges to maintaining the effect of free healthcare policies, such as the *gratuité*. Besides, the effect of free healthcare policy may vary depending on contextual factors, such as geographical location and socioeconomic conditions. Future research should explore how the effects of *gratuité* differ across contextual factors, including geographical areas and socioeconomic groups in Burkina Faso.

## Supplementary Material

czae077_Supp

## Data Availability

The data underlying this article were provided with permission from the Ministry of Health, Burkina Faso. Data will be shared on request to the corresponding author with permission of the Ministry of Health.

## Reflexivity statement

The authorship team comprises five female and four male researchers. Four co-authors are from low- and middle-income countries (LMICs), with two specifically from Burkina Faso. The remaining authors build on an extensive research and policy experience in Burkina Faso and the surrounding region. With members at various levels of seniority, the team possesses diverse expertise in healthcare, health economics, health financing, statistics, health policy and health system research in LMICs.
