# Supplementary material for: How do free healthcare policies impact utilization of maternal and child health services in fragile settings? Evidence from a controlled interrupted time series analysis in Burkina Faso
Source: Health Policy Plan. 2024 Aug 24;39(9):891–901. doi: 10.1093/heapol/czae077 (PMC11474610; doi:10.1093/heapol/czae077)
Supplement: czae077_Supp [file czae077_supp.zip › FHC_BF_ITSA_supplementary.docx]

## Appendices

### Appendix A: Model estimation of *gratuité* using single ITSA

Table A.1: Model estimation of single ITSA for facility-based delivery

|  | Estimate (%) | 95% CI | p-value |
| --- | --- | --- | --- |
| Initial level | 71.05 | 66.11, 75.99 | <0.001*** |
| Pre-policy trend | -0.05 | -0.24, 0.14 | 0.603 |
| **Effect of *gratuité:*** |  |  |  |
| Level change | 3.95 | -1.72, 9.61 | 0.176 |
| Trend change | -0.34 | -0.79, 0.1 | 0.137 |
| Trend change (square) | 0.01 | 0, 0.01 | 0.058 |

Table A.2: Model estimation of single ITSA for children <5 years

|  | Estimate (rate) | 95% CI | p-value |
| --- | --- | --- | --- |
| Initial level | 113.14 | 84.07, 142.2 | <0.001 *** |
| Pre-policy trend | 0.47 | -0.53, 1.47 | 0.360 |
| **Effect of *gratuité:*** |  |  |  |
| Level change | 101.98 | 73.1, 130.86 | <0.001 *** |
| Trend change | -1.18 | -2.32, -0.03 | 0.047 * |

### Appendix B: Comparison of fitting of the models with various adjustments

|  | **ARMA selected** | **Residual standard error** | **AIC** | **BIC** | **Loglik** |
| --- | --- | --- | --- | --- | --- |
| **Facility-based delivery** |  |  |  |  |  |
| Simple linear regression | - | 7.784 | 1509.307 | 1539.684 | -745.653 |
| Generalized least squares (GLS) | P=12, q=0 | 7.642 | 1406.058 | 1476.939 | -682.029 |
| GLS with seasonality-adjusted | P=12, q=0 | 6.541 | 1370.542 | 1478.551 | -653.271 |
| GLS with seasonality-adjusted and quadratic term **(Chosen model)** | **P=12, q=0** | **6.384** | **1368.283** | **1483.042** | **-650.141** |
| **Curative care for children <5 years** |  |  |  |  |  |
| Simple linear regression | - | 40.420 | 2098.080 | 2127.944 | -1040.040 |
| Generalized least squares (GLS) | p=2, q=1 | 39.964 | 1906.358 | 1946.176 | -941.179 |
| GLS with seasonality-adjusted **(Chosen model)** | **p=2, q=1** | **25.343** | **1849.508** | **1925.825** | **-901.754** |
| GLS with seasonality adjusted and quadratic term | p=2, q=1 | 25.450 | 1852.990 | 1935.943 | -901.495 |

### Appendix C: Results of the unselected models: Linear model for facility-based delivery and Quadratic model for children <5 years

Table C.1: Model estimation of *gratuité* effect on facility-based delivery utilization (linear form)

| **Facility-based delivery (ANC1 as control)** | **Estimates (%)** | **95% CI** | **p-value** |
| --- | --- | --- | --- |
| **Initial level:** |  |  |  |
| ANC1 | 84.79 | 77.36, 92.22 | <0.001 *** |
| Difference between facility-based delivery and ANC1 | -7.81 | -15.94, 0.32 | 0.061 |
| **Before policy trend:** |  |  |  |
| ANC1 | -0.19 | -0.41, 0.02 | 0.082 |
| Difference between facility-based delivery and ANC1 | 0.18 | -0.13, 0.48 | 0.262 |
| **Post-policy period (ANC1):** |  |  |  |
| Level change | 2.01 | -3.78, 7.79 | 0.497 |
| Trend change | 0.12 | -0.15, 0.38 | 0.400 |
| **Effect of *gratuité (*Facility-based delivery):** |  |  |  |
| Level change | -2.14 | -10.28, 6 | 0.607 |
| Trend change | -0.11 | -0.49, 0.26 | 0.554 |

Table C.2: Model estimation of the policy effects on the utilization of curative care for children <5 years (quadratic form)

| **Curative care for children <5 years**  **(Children 5-14 years as control)** | **Estimates**  (rate*) | **95% CI** | **p-value** |
| --- | --- | --- | --- |
| **Initial level:** |  |  |  |
| Children 5-14 years | 20.03 | 3.51, 36.54 | 0.018 * |
| Difference between children <5 years and 5-14 years | 102 | 86.12, 117.88 | <0.001 *** |
| **Pre-policy trend:** |  |  |  |
| Children 5-14 years | 0.12 | -0.39, 0.63 | 0.640 |
| Difference between children <5 years and 5-14 years | 0.06 | -0.65, 0.77 | 0.874 |
| **Post-policy period (Children 5-14 years):** |  |  |  |
| Level change | 7.73 | -16.1, 31.56 | 0.526 |
| Trend change | 0.003 | -1.36, 1.37 | 0.996 |
| Trend change (square) | -0.003 | -0.03, 0.02 | 0.845 |
| **Effect of *gratuité (Children <5 years)*:** |  |  |  |
| Level change | 107.79 | 76.54, 139.05 | <0.001 *** |
| Trend change | -0.8 | -2.42, 0.82 | 0.335 |
| Trend change (square) | -0.002 | -0.03, 0.03 | 0.891 |

### Appendix D: Sensitivity analyses of gratuité on the utilization of facility-based delivery: model using ANC4 as a control

| ANC4 as control | Estimates (%) | 95% CI | p-value |
| --- | --- | --- | --- |
| **Initial level:** |  |  |  |
| ANC4 | 22.1 | 16.55, 27.64 | <0.001 *** |
| Difference between delivery and ANC4 | 50.55 | 44.87, 56.23 | <0.001 *** |
| **Pre-policy trend:** |  |  |  |
| ANC4 | 0.2 | 0.03, 0.36 | 0.018 * |
| Difference between delivery and ANC4 | -0.22 | -0.45, 0.01 | 0.062 |
| Post-policy period (ANC4): |  |  |  |
| Level change | -0.11 | -5.36, 5.13 | 0.966 |
| Trend change | -0.26 | -0.63, 0.11 | 0.173 |
| Trend change (squared) | 0.003 | -0.001, 0.01 | 0.193 |
| **Effect of *gratuité (Facility-based delivery)*:** |  |  |  |
| Level change | 3.63 | -3.72, 10.97 | 0.335 |
| Trend change | -0.1 | -0.63, 0.42 | 0.694 |
| Trend change (squared) | 0.002 | -0.004, 0.01 | 0.495 |

### Appendix E: Sensitivity analyses of gratuité on the utilization of facility-based delivery: Model 1 - with April 2016 as an interruption, Model 2 - excluding months with large missing in 2019, Model 3 - excluding 2021 data

| ANC1 as control | Model 1 | | | Model 2 | | | Model 3 | | |
| --- | --- | --- | --- | --- | --- | --- | --- | --- | --- |
|  | Estimates (%) | 95% CI | p-value | Estimates (%) | 95% CI | p-value | Estimates (%) | 95% CI | p-value |
| **Initial level:** |  |  |  |  |  |  |  |  |  |
| ANC1 | 84.69 | 77.79, 91.59 | <0.001 *** | 82.93 | 77.25, 88.6 | <0.001 *** | 85.75 | 79.41, 92.08 | <0.001 *** |
| Difference between delivery and ANC1 | -7.73 | -14.74, -0.73 | 0.032 * | -6.48 | -12.39, -0.58 | 0.033 * | -7.44 | -12.85, -2.03 | 0.008 ** |
| **Pre-policy trend:** |  |  |  |  |  |  |  |  |  |
| ANC1 | -0.16 | -0.37, 0.04 | 0.122 | -0.15 | -0.32, 0.02 | 0.079 | -0.18 | -0.34, -0.03 | 0.024 * |
| Difference between delivery and ANC1 | 0.18 | -0.11, 0.46 | 0.229 | 0.12 | -0.12, 0.35 | 0.324 | 0.16 | -0.06, 0.38 | 0.149 |
| **Post-policy period (ANC1):** |  |  |  |  |  |  |  |  |  |
| Level change | 2.94 | -3.25, 9.12 | 0.353 | 3.84 | -2, 9.68 | 0.199 | 4.76 | -1.15, 10.68 | 0.116 |
| Trend change | -0.17 | -0.6, 0.26 | 0.445 | -0.23 | -0.64, 0.19 | 0.282 | -0.21 | -0.65, 0.23 | 0.346 |
| Trend change (square) | 0.004 | -0.007, 0.01 | 0.134 | 0.005 | -0.001, 0.01 | 0.097 | 0.004 | -0.003, 0.01 | 0.255 |
| **Effect of *gratuité (Facility-based delivery)*:** |  |  |  |  |  |  |  |  |  |
| Level change | -1.68 | -10.39, 7.02 | 0.705 | -1.69 | -9.84, 6.46 | 0.686 | -3.9 | -12.23, 4.43 | 0.360 |
| Trend change | -0.12 | -0.73, 0.49 | 0.697 | 0.01 | -0.57, 0.59 | 0.972 | 0.19 | -0.43, 0.8 | 0.553 |
| Trend change (square) | -0.0001 | -0.01, 0.01 | 0.976 | -0.001 | -0.01, 0.01 | 0.743 | -0.01 | -0.02, 0 | 0.233 |

### Appendix F: Sensitivity analyses of *gratuité* on the utilization of curative care for children <5 years: Model 1 - with April 2016 as an interruption, Model 2 - excluding months with large missing in 2019, Model 3 - excluding 2021 data

| Children 5-14 years as control | Model 1 | | | Model 2 | | | Model 3 | | |
| --- | --- | --- | --- | --- | --- | --- | --- | --- | --- |
|  | Estimates (rate) | 95% CI | p-value | Estimates (rate) | 95% CI | p-value | Estimates (rate) | 95% CI | p-value |
| **Initial level:** |  |  |  |  |  |  |  |  |  |
| Children 5-14 years | 22.21 | -0.31, 44.72 | 0.055 | 21.63 | 6.89, 36.36 | 0.005 ** | 22.81 | 6.6, 39.02 | 0.006 ** |
| Difference between children <5 years and 5-14 years | 101.39 | 74.71, 128.06 | <0.001 *** | 104.38 | 91.49, 117.28 | <0.001 *** | 103.38 | 88.12, 118.64 | <0.001 *** |
| **Pre-policy trend:** |  |  |  |  |  |  |  |  |  |
| Children 5-14 years | 0.05 | -0.78, 0.88 | 0.910 | 0.12 | -0.28, 0.53 | 0.553 | 0.12 | -0.36, 0.59 | 0.632 |
| Difference between children <5 years and 5-14 years | 0.05 | -1.11, 1.22 | 0.928 | -0.08 | -0.64, 0.49 | 0.791 | -0.03 | -0.71, 0.64 | 0.920 |
| **Post-policy period (Children 5-14 years):** |  |  |  |  |  |  |  |  |  |
| Level change | 9.59 | -14.26, 33.45 | 0.431 | 6.26 | -6.85, 19.37 | 0.351 | 9.38 | -5.42, 24.18 | 0.216 |
| Trend change | -0.03 | -0.96, 0.9 | 0.950 | 0.06 | -0.33, 0.46 | 0.751 | -0.13 | -0.58, 0.32 | 0.565 |
| **Effect of *gratuité (Children <5 years)*:** |  |  |  |  |  |  |  |  |  |
| Level change | 99.78 | 66.96, 132.6 | <0.001 *** | 116.94 | 99.57, 134.31 | <0.001 *** | 115.57 | 94.72, 136.42 | <0.001 *** |
| Trend change | -0.66 | -1.94, 0.62 | 0.310 | -1.02 | -1.56, -0.49 | <0.001 *** | -1 | -1.63, -0.36 | 0.002 ** |

### Appendix G: Sensitivity analyses of gratuité on the utilization of facility-based delivery after controlling for COVID-19

| **Facility-based delivery (ANC1 as control)** | **Estimates (%)** | **95% CI** | **p-value** |
| --- | --- | --- | --- |
| **Initial level:** |  |  |  |
| ANC1 (β0) | 85.82 | 83.72, 87.93 | <0.001 *** |
| Difference between delivery and ANC1 (β5) | -6.91 | -9.89, -3.94 | <0.001 *** |
| **Pre-policy trend:** |  |  |  |
| ANC1 (β1) | -0.18 | -0.26, -0.09 | <0.001 *** |
| Difference between delivery and ANC1 (β6) | 0.14 | 0.02, 0.27 | 0.023 * |
| **Post-policy period (ANC1):** |  |  |  |
| Level (β2) | 1.9 | -1.93, 5.73 | 0.333 |
| Trend (β3) | 0.19 | -0.12, 0.51 | 0.236 |
| Trend (square) (β4) | -0.01 | -0.01, 0.0007 | 0.082 |
| **Effect of *gratuité* (Facility-based delivery):** |  |  |  |
| Level change (β7) | -4.88 | -10.3, 0.54 | 0.079 |
| Trend change (β8) | 0.4 | -0.04, 0.85 | 0.079 |
| Trend change (square) (β9) | -0.01 | -0.02, -0.001 | 0.024 * |
| **Effect of COVID-19** |  |  |  |
| COVID-19 level change | 2.69 | -4.23, 9.62 | 0.447 |
| COVID-19 trend change | 1.51 | 0.35, 2.68 | 0.012 * |
| COVID-19 trend (square) | -0.03 | -0.08, 0.02 | 0.233 |
| COVID-19 level * delivery | -0.49 | -10.28, 9.3 | 0.922 |
| COVID-19 trend change * delivery | 0.48 | -1.17, 2.13 | 0.569 |
| COVID-19 trend change (square) * delivery | 0.02 | -0.05, 0.1 | 0.517 |

### Appendix H: Sensitivity analyses of gratuité on the utilization of curative care for children <5 years after controlling for COVID-19

| **Curative care for children <5 years**  **(Children 5-14 years as control)** | **Estimates (rate)** | **95% CI** | **p-value** |
| --- | --- | --- | --- |
| **Initial level:** |  |  |  |
| Children 5-14 years (β0) | 19.26 | 3.85, 34.67 | 0.015 * |
| Difference between children <5 years and 5-14 years (β4) | 103.23 | 90.44, 116.02 | <0.001 *** |
| **Pre-policy trend:** |  |  |  |
| Children 5-14 years (β1) | 0.1 | -0.3, 0.5 | 0.620 |
| Difference between children <5 years and 5-14 years (β5) | 0.002 | -0.56, 0.57 | 0.994 |
| **Post-policy period (Children 5-14 years):** |  |  |  |
| Level change (β2) | 10.5 | -3.23, 24.24 | 0.136 |
| Trend change (β3) | -0.15 | -0.56, 0.26 | 0.476 |
| **Effect of *gratuité* (Children <5 years):** |  |  |  |
| Level change (β6) | 107.94 | 88.59, 127.3 | <0.001 *** |
| Trend change (β7) | -0.69 | -1.26, -0.11 | 0.02 * |
| **Effect of COVID-19** |  |  |  |
| COVID-19 level change | -3.51 | -47.51, 40.48 | 0.876 |
| COVID-19 trend change | 1.09 | -6.2, 8.39 | 0.769 |
| COVID-19 level change * Children <5 years | -33.71 | -82.85, 15.43 | 0.180 |
| COVID-19 trend change * Children <5 years | 1.45 | -5.95, 8.85 | 0.701 |
